# A Case of Bronchial Diverticulum With Progressive Enlargement and Abscess Formation Successfully Managed by Endobronchial Ultrasound‐Guided Transbronchial Needle Aspiration and Antibiotic Therapy

**DOI:** 10.1002/rcr2.70251

**Published:** 2025-06-22

**Authors:** Hikaru Aoki, Masaki Aikawa, Yoshitomo Kushima, Jiro Watanabe, Toshiyuki Shima, Yoju Kameyama

**Affiliations:** ^1^ Department of Thoracic Surgery Ashikaga Red Cross Hospital Ashikaga Japan; ^2^ Department of Medicine Ashikaga Red Cross Hospital Ashikaga Japan

**Keywords:** airway infection, bronchial diverticulum, cystic airway lesion, endobronchial ultrasound‐guided transbronchial needle aspiration, tracheal diverticulum

## Abstract

Bronchial diverticulum (BD) is a rare airway abnormality that rarely enlarges significantly or becomes infected. We report a case of an 80‐year‐old male with a 15‐year history of asymptomatic BD that gradually increased in size and eventually developed into an abscess. Chest computed tomography revealed a cystic lesion adjacent to the left main bronchus with internal fluid and surrounding inflammation. Endobronchial ultrasound‐guided transbronchial needle aspiration (EBUS‐TBNA) enabled safe aspiration and identification of alpha‐hemolytic *Streptococcus* despite the absence of a visible bronchial opening. The infection was successfully managed with antibiotics alone, thereby avoiding surgical intervention. This case highlights the potential for long‐term asymptomatic BD to develop infection and demonstrates the diagnostic utility of EBUS‐TBNA for lesions adjacent to vital structures. However, further studies are needed to clarify infection risk factors and optimal management strategies for BD.

## Graphical abstract

This report describes an 80‐year‐old male with bronchial diverticulum that remained asymptomatic for over 15 years before gradually enlarging and developing into an infected abscess. Endobronchial ultrasound‐guided transbronchial needle aspiration (EBUS‐TBNA) successfully identified alpha‐hemolytic *Streptococcus* and enabled targeted antibiotic treatment without surgical intervention. This case indicates that late complications may develop in long‐standing bronchial diverticulum and demonstrates the benefits of EBUS‐TBNA for managing complicated lesions adjacent to vital structures.

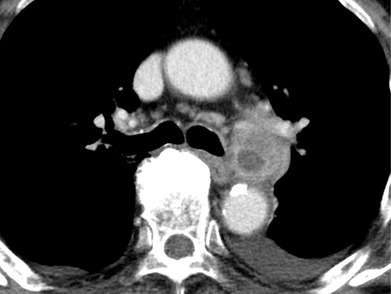

## 1 Introduction

Bronchial diverticulum (BD) is a rare congenital or acquired outpouching of the bronchial wall that is increasingly detected incidentally with the widespread use of high‐resolution chest computed tomography (CT) [1]. Compared to tracheal diverticulum, BD is reported far less frequently; however, it remains uncertain whether this reflects a genuinely lower incidence or is owing to under‐recognition and underreporting. Consequently, the clinical significance, natural history and optimal management of BD are poorly understood owing to the limited number of published cases. Most BDs are asymptomatic and typically managed conservatively [2]. However, robust evidence regarding the long‐term risks of enlargement, infection, or other complications is lacking and no consensus exists regarding the indications for intervention.

Here, we present a rare case of BD that remained asymptomatic for over 15 years before gradually enlarging and ultimately becoming infected, resulting in abscess formation. This case highlights the potential for late complications in BD and underscores the diagnostic and therapeutic utility of endobronchial ultrasound‐guided transbronchial needle aspiration (EBUS‐TBNA) for the management of infected lesions.

## 2 Case Report

**FIGURE 1:**
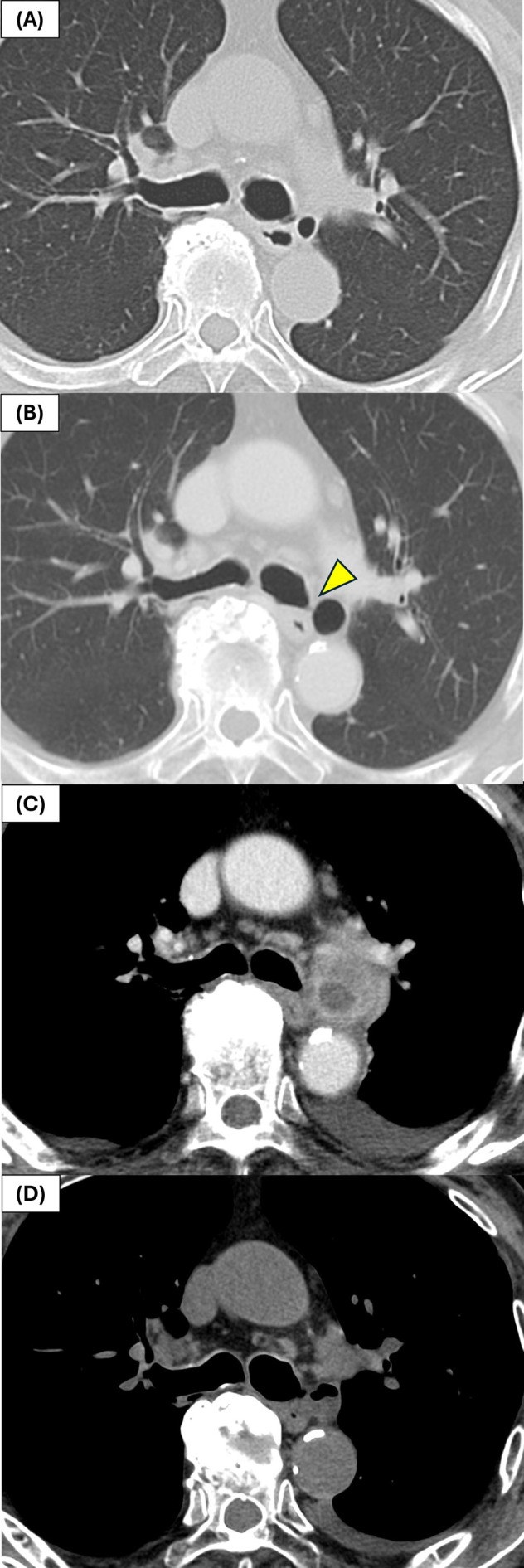
Serial chest computed tomography (CT) images demonstrating the progression of bronchial diverticulum (BD). (A) In 2013, an 8‐mm BD was observed adjacent to the left main bronchus. (B) In 2020, the BD had enlarged to 14 mm, with clear evidence of communication with the bronchial lumen (yellow arrow). (C) In 2024, the diverticulum had further increased in size to 18 mm, with internal fluid accumulation and increased attenuation of the surrounding adipose tissue, indicating inflammation. (D) One‐month post‐treatment, the size of the diverticulum remained unchanged, with a mixture of fluid and gas inside, but the attenuation of the surrounding adipose tissue had returned to normal.

An 80‐year‐old male with a five pack‐year smoking history and history of asthma presented with fever in 2024. Laboratory investigations revealed mildly elevated C‐reactive protein level (5.24 mg/dL). Chest CT revealed a cystic lesion with internal fluid accumulation located between the left main bronchus and aortic arch, accompanied by increased attenuation of the surrounding adipose tissue. A retrospective review of prior imaging findings revealed a gas‐filled cystic lesion in the same location, consistent with BD. The BD diameter was 8 mm in 2009, increased to 14 mm in 2020 with evidence of communication with the bronchus, and further increased to 18 mm by 2024 (Figure 1).

**FIGURE 2:**
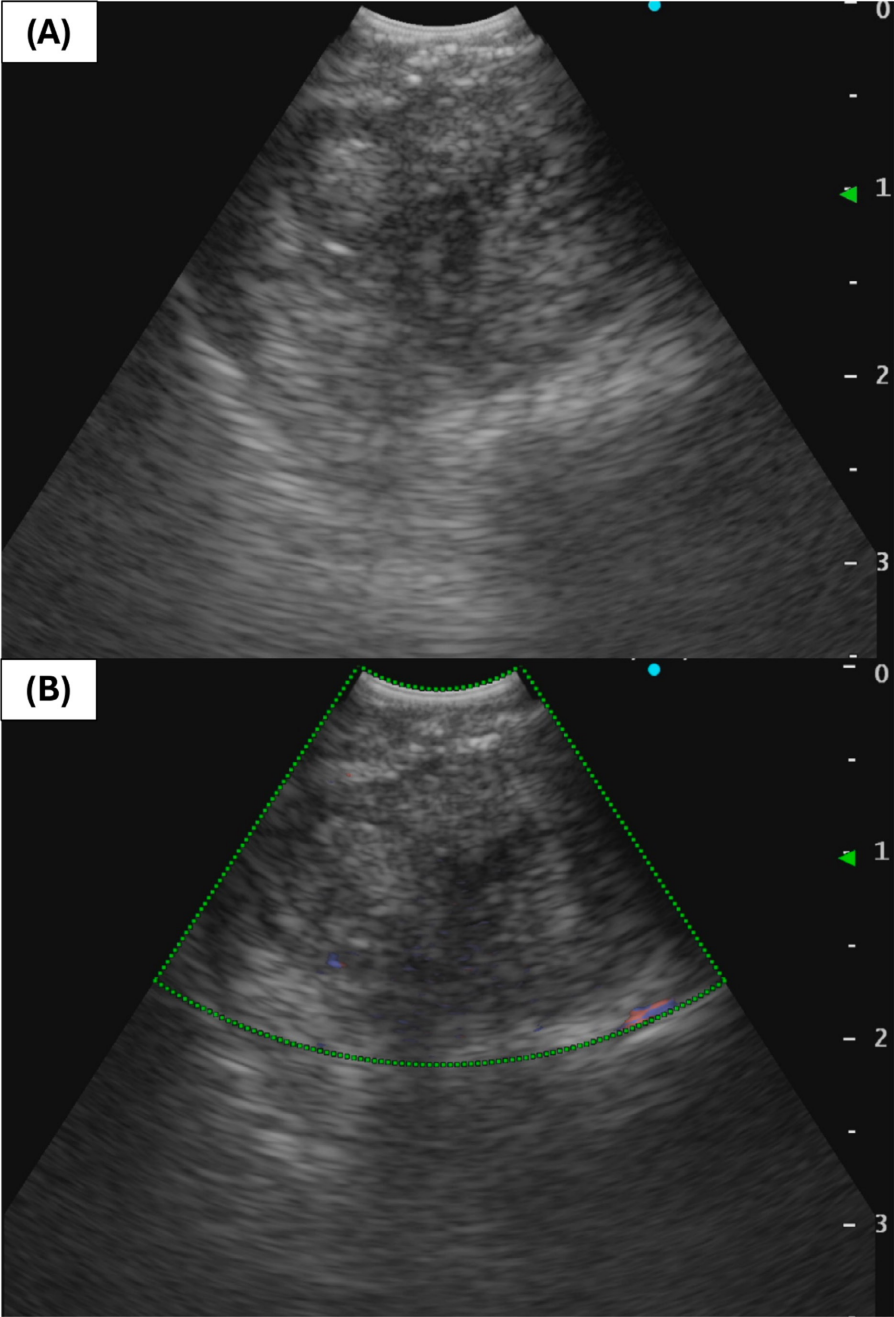
Endobronchial ultrasonography (EBUS) findings of the bronchial diverticulum. (A) EBUS revealing a hypoechoic lesion adjacent to the left main bronchus. (B) EBUS showing no detectable blood flow within the lesion.

Bronchoscopy was performed to identify the causative organism; however, no obvious opening of the BD was observed in the bronchial lumen and no other abnormal findings were noted. The BD was localised near the junction between the cartilaginous and membranous portions of the left main bronchus using EBUS. EBUS revealed a hypoechoic lesion with no detectable blood flow (Figure 2). Under EBUS guidance, a 22‐gauge needle was used to aspirate whitish purulent fluid from the lesion. Culture of the aspirated specimen showed alpha‐hemolytic *Streptococcus*. Antibiotic therapy led to resolution of inflammation. One month later, follow‐up CT showed that the BD contained both fluid and gas, but attenuation of the surrounding adipose tissue returned to normal.

## 3 Discussion

BD is an uncommon disease, with most cases identified accidentally on high‐resolution chest CT and remaining clinically silent throughout the patient's lifetime [2]. The true prevalence and natural history of BD are not well established, primarily owing to the absence of large‐scale epidemiological studies. Although most BDs are asymptomatic, this case demonstrates that late‐onset complications such as infection and abscess formation can occur even after prolonged periods of quiescence. This underscores the importance of long‐term monitoring in patients with BD, particularly in those with an increasing diverticular size or evidence of communication with the bronchial lumen.

To our knowledge, to date, there is no consensus regarding the optimal management of BD, and robust evidence to guide clinical decision‐making is lacking. Most asymptomatic cases are managed conservatively, with interventions reserved for symptomatic or complicated lesions [3, 4]. However, the risk factors predisposing patients to infections or other adverse outcomes remain unclear. Our case is notable for the gradual enlargement of BD over more than a decade, culminating in infection and abscess formation. This temporal progression suggests that diverticular growth and bronchial communication may be important determinants of complication risk. However, further studies are required to validate these observations.

Diagnostic and therapeutic approaches for complicated BD have not yet been standardised. In this case, EBUS‐TBNA proved invaluable, enabling the safe and effective aspiration of purulent material for microbiological analysis. Although EBUS‐TBNA is well‐established for the evaluation of mediastinal lymphadenopathy and peribronchial masses, its application in the management of infected BD has rarely been reported. Our results demonstrate that EBUS‐TBNA can serve as a minimally invasive alternative to surgical intervention, facilitating targeted antimicrobial therapy and rapid recovery.

Given the limited data on BD, prospective multicentre studies and long‐term registries are needed to better characterise the natural history of BD, identify risk factors for complications and establish evidence‐based management guidelines. In particular, research should focus on the prognostic significance of diverticular size, presence of bronchial communication and patient comorbidities in predicting adverse outcomes. Until such data become available, clinicians should maintain a high index of suspicion for late complications in patients with BD, particularly those with radiological progression or new‐onset symptoms.

## Author Contributions

Hikaru Aoki contributed to the interpretation of the data and drafting of the manuscript. All authors critically revised the manuscript for important intellectual content and approved the final version.

## Consent

The authors declare that written informed consent was obtained for the publication of this manuscript and accompanying images using the form provided by the journal.

## Conflicts of Interest

The authors declare no conflicts of interest.

## Data Availability

Data sharing not applicable to this article as no datasets were generated or analysed during the current study.
